# A Survey of Complementary and Alternative Medicine Use in Cancer Patients Treated with Radiotherapy in Thailand

**DOI:** 10.1155/2012/670408

**Published:** 2012-02-23

**Authors:** Putipun Puataweepong, Nongnuj Sutheechet, Panjachat Ratanamongkol

**Affiliations:** ^1^Radiotherapy and Oncology Unit, Department of Radiology, Faculty of Medicine, Ramathibodi Hospital, Mahidol University, Rajtawee, Bangkok 10400, Thailand; ^2^Department of Anesthesiology, Nopparat Ratchthani Hospital, Kannayao, Bangkok 10230, Thailand; ^3^Department of Paediatrics, Bhumibol Adulyadej Hospital, Saimai, Bangkok 10220, Thailand

## Abstract

*Introduction*. Use of complementary and alternative medicine (CAM) in cancer patients is increasingly acceptable worldwide, but most of the studies were surveyed from developed countries. In this study, we evaluated the first and large cohort of cancer patients with CAM use in Thailand. *Materials and Methods*. A self-administered questionnaire was completed by 248 cancer patients attending outpatient radiotherapy unit at Ramathibodi Hospital. *Results*. The prevalence of CAM use was 60.9%. The most frequently used CAM were dietary/vitamin supplements (56.9%). Independent predictors of CAM use were high income (*P* < 0.001) and cancer type (*P* = 0.019). About half of the patients (51%) reported positive effects from CAM use. Nevertheless, 9.4% of the patient also reported side effects. The majority of patients (58.3%) did not disclose their use of CAM to their doctors because they felt that it was not necessary for doctors to know (65.9%). The average spending for CAM use was 200 USD/month (range, 10–1,000). *Conclusion*. Although the cost for CAM is relatively expensive, the prevalence of CAM use in cancer patients in Thailand is high particularly, in patients with higher income. Therefore, all clinical oncologists should be concerned about the use of CAM during evaluation of the cancer patients.

## 1. Introduction

Cancer is the major cause of death in most countries throughout the world. The main standard or conventional therapies such as surgery, chemotherapy, radiotherapy, and hormone therapy usually cause many adverse effects. Complementary and alternative medical (CAM) practices have become increasingly popular worldwide and many cancer patients have turned to CAM with hope of finding a cure to their illness, as well as to make them feel better. The National Center for Complementary and Alternative Medicine (NCCAM) defines CAM as a group of diverse medical and healthcare systems, practices, and products that are not considered to be part of conventional medicine [1]. The prevalence of CAM use in cancer patients is frequently high and estimated to be from 30% to 90% [2–7]. The update systematic review [3] was the surveyed studies published from 18 countries in Australia, Canada, Europe, New Zealand, and the United States. From this study, the combined prevalence for current use of CAM in cancer patients was 40%. The highest was in the United States and the lowest in Italy and the Netherlands. This metaanalysis also suggested an increase in CAM use from an estimated 25% in the 1970s and 1980s to more than 32% in the 1990s and to 49% after 2000. Nevertheless, most of studies for CAM use in cancer patients usually came from western and developed countries. So far, very few studies have described the use of CAM in developing countries. To date in South East Asia including Thailand, the rate of CAM use among cancer patients is unknown. The use of traditional herbs and remedies in our country is, however, well known and relatively common. We evaluated the first and large cohort of cancer patients with CAM use in Thailand. Understanding CAM use among cancer patients may provide insight into the motivations behind such use and, therefore, the degree to which conventional medical care has not met the needs of cancer patients. Thus, the aims of this study were to determine the prevalence and pattern of CAM use, reason for using CAM, the perceived effectiveness as well as their communication with doctors about its use.

## 2. Materials and Methods

The study design was a descriptive cross-sectional study conducted at the radiotherapy outpatient clinic at Ramathibodi Hospital, Bangkok, Thailand. It was approved by the Ethics Committee on Human Experimentation of the hospital.

### 2.1. The Questionnaire

The questionnaire used in this study was the newly developed self-administering questionnaire, because currently there is no related and proper questionnaire developed in Thailand. After an extensive literature reviews on CAM in cancer patients, the 21-item questionnaire was developed on the basis of the standard questionnaire development (see the appendix).

### 2.2. Study Subjects

All cancer patients attending the radiotherapy outpatient clinic of Ramathibodi Hospital from 1 June to 30 July 2011 were recruited into the study. The inclusion criteria were all of 18-year and older patients with diagnosis of cancer within 3 years, writing ability in Thai, and willingness to participate in this study.

### 2.3. Data Collection

All patients who met the inclusion criteria during study period were invited to participate. Information about the research was given verbally to each patient; those who gave consent then filled in the questionnaires. The participants used 10–15 minutes to complete the questionnaire while they were waiting at the outpatient clinic to be seen by their physicians. Physicians who were in any way involved in the treatment of each patient were not present during the administration of the questionnaire. On completion, the patients either put the questionnaire in a box or handed it to the researcher assistant.

### 2.4. Statistical Analysis

The demographic characteristic data were calculated by descriptive statistics. Categorical data were described with frequency and percentage and compared by using chi-square. Continuous data were reported with mean and range and compared by using student's *t*-test. All analyses were performed using SPSS software version 16.0.

## 3. Results

There were 248 cancer patients participating in this study. One hundred and fifty-one (60.9%) of the total participants reported having used at least one CAM since their diagnosis of cancer.  Table 1 shows the demographic characteristics of CAM users and non-CAM users. There were no significant differences in the proportion of CAM users by gender, age, marital status, religion, education level, occupation, cancer type, or cancer staging. There were, however, significant differences in the proportion of cancer patients using CAM by income achievement (*P* = 0.001) and by the cancer type (*P* = 0.019). The patients with a higher income were more likely to use CAM than those with a lower income. With regard to the cancer type, the highest prevalence rate of CAM use was by those with malignant brain tumor, followed by those with lung cancer, and those with genitourinary cancer. The lowest rates of CAM use were observed in gastrointestinal cancer patients. The CAM products/therapies that were used are shown in Table 2. The most common CAM was dietary/vitamin supplement followed by dietary adjustment, meditation, herbal medicine, and massage, respectively.

 Most patients were using CAM because as they wanted to counteract suffering symptoms from the cancer or medical treatment (33.1%), to directly fight the disease or decrease the tumor (31.1%), to assist conventional treatment (25.2%), to improve physical well-being (17.2%), to improve emotional well-being or provide hope (11.3%), and as well as to do everything possible to fight the disease (3.3%).

 About half of the patients reported positive effects from CAM use including good effect (20%) and moderate effect (31.0%), while 10.3% of patients reported no effect from CAM use. Nevertheless, 38.6% of patients were uncertain about their effect. Fourteen patients (9.4%) reported side effects from the CAM therapy they had used, most of which seemed to be related to ingesting herbs or minerals and massage. These side effects included decrease in appetite (5 cases), diarrhea (3 cases), exhaustion (3 cases), nausea-vomiting (2 cases), gastric discomfort (2 cases), constipation (1 case), abnormal menstruation (1 case), and muscle sprain (1 case). Moreover, two patients complained about the cost of their CAM use.

The majority of CAM users (58.2%) did not disclose the use of CAM to their medical doctors, the most common reasons were that it was not necessary for the doctors to know (65.9%), or the doctors never asked (40.9%) or the doctors would disapprove of it (33.0%). Sixty-three patients (41.7%) had told their doctors that they were using CAM. 39.7% of doctors responded favorably, 33.3% of doctors were against it, and 27% of doctors did not offer any opinion about CAM uses. Reasons for disclosure of CAM use to their doctors were “the doctor asked” (37.1%), “the doctor should know” (20.5%), and “wanted to know doctor's opinion about CAM use” (2.0%).

Patients were asked how much on average they spent on CAM in one month. Only 58 out of 151 patients reported expenses (38.4%). The average spending was 200 USD/month, (with the range of 10–1,000). However, 3 patients reported that they used herbal medicine which they planted for their own use; therefore, they had no expenditure for CAM. Likewise, one patient had relatives massage for him and had no expenditure.

## 4. Discussion

To our knowledge, this is the first study of the use of CAM by patients with a variety of cancers in Thailand, and it is one of the few representative studies available about the use of CAM in cancer patient in Asia. The use of CAM by cancer patients is very common and varies widely among populations. The update systematic review from Horneber et al. [3] that surveyed a total of 152 studies from 18 countries in the western world such as Australia, Canada, Europe, New Zealand, and the United States reported that the prevalence for current use of CAM across all studies was 40%. Regarding the prevalence of CAM use in Asian countries, there is very few study reports, but the prevalence of CAM use seems to be higher than that from the western countries. For the example, the prevalence of CAM use ranged from 54% to 61% in Turkey [4, 8], 64% in Malaysia [5], 60.9% in Palestine [9], 55% in Singapore [10], and 93.4% in China [11]. The rate of 60.9% that we found in this study is quite similar with the papers from Asian countries but higher than that of the study from Western countries. The higher prevalence rate in our study and in Asian countries may be explained by multiple factors such as traditional culture, religious beliefs, the cost of conventional treatment or the methodology, and the instrument used to collect the data. Sociodemographic factors that appear to be related to CAM use are younger age, higher education, higher income, married status, involvement in a support group, and health insurance [12]. In the present study, it was found that people from higher income used CAM more frequently. It was also interesting to see the prevalence rates of CAM use among different cancer types and stages. Despite suggestions from the literature that CAM applications were significantly higher in the group with advanced diseases and recurrent diseases [13], the present study showed that brain and lung cancer patients used CAM therapies significantly more often than any other cancer types. The possibility of the higher prevalence in both cancer types might be because both of these diagnostic categories are characterised by poor prognosis and a rapid physical decline, often with metastasis present, and such patients may have little hope from conventional treatments, thus turning to CAM as an additional intervention to improve their lives. The role of CAM may be important, not only because it increases hope and optimism, but also improves quality of life and helps manage symptoms, especially in terminal illness; however relevant data in cancer patients are almost nonexistent to date. However, some of the results in this subgroup analysis should be viewed with caution, as only a small number of patients participated in some of the diagnostic categories.

There are many types of CAM use worldwide. The most popular CAM uses were dietary supplements, herbs and botanicals, and relaxation techniques/meditation [2, 12, 14, 15]. In our surveyed population, the most frequently used CAM was dietary and vitamin supplement, followed by dietary adjustment. The choice of the specific CAM treatment used is based primarily on individual patient complaints and problems, which may explain the discrepancies among the studies. Furthermore, the stage of the cancer and the approval of the patient's physician may contribute to determining the type of CAM preferred by the patient. In cases of advanced cancer, spiritual or relaxation therapies may be the most appropriate complementary treatments, whereas homeopathy or acupuncture may be the more popular treatments of choice in earlier stages of cancer or in other chronic diseases. Additional parameters that may affect treatment choice are different cultural norms, backgrounds, and religious beliefs.

 The major expectation of the patients in this study was “counteract suffering symptoms from the disease or medical treatment.” Since many of these therapies used are “complementary” in nature (such as aromatherapy, massage, meditation, and others), we may not need to prove their effectiveness before using them. As patients are demanding such therapies, they are low-risk therapies and patients feel good after their use. Such therapies may have a great role to play, especially in the palliative care setting, where the goal is not cure but rather improvement in quality of life. Patient satisfaction can be an appropriate end point outcome for evaluation in this setting rather than clinical outcome.

For the positive and negative effects from its use, half of the patients seemed to be satisfied with the use of CAM, for they reported good or moderate benefit from it. A wide range of reasons may contribute to the use of CAM, and perhaps the concept of “hope” is fundamental in each one of these reasons. More than 30% of the patients used CAM therapies to directly fight the cancer or to decrease the tumor burden. It is interesting to see that <5% of the patients used CAM following the recommendation of their physician. These findings coincide with findings from the other previous studies [5–7, 16–19] and perhaps are reflecting the disapproval of CAM therapies by the medical community or the lack of information within the medical community about available and effective CAM therapies. Most patients reported no adverse reactions to CAM. However, the potential for harmful drug: CAM product interactions exists. There was a report showing that the use of CAM is also associated with a significant delay in cancer treatment [20].

Almost 60% of cancer patients who used CAM since the diagnosis of cancer did not disclose the use of their CAM therapies to their doctors. The main reasons for nondisclosure were: “It was not necessary for the doctor to know,” 41% of patients reported that “their doctors did not ask,” and one-third of the patients feared disapproval from their doctors. These findings are consistent with those of other investigators [5–7, 16–19].

 In our survey, when patients consulted their doctors, almost 40% of them were told that they were free to continue using CAM but one-third of the patients were told to stop. These figures were also similar to the results in a previous study of clinical oncologists [21]. It appears that a difficult situation for many oncologists emerges because of their lack of scientific information on CAM. However, physicians should acknowledge that 40.9% of patients did not inform their physicians of their CAM use because their doctors did not ask them. These results indicate that better patient-physician communication and more reliable information on CAM products are needed.

It has been suggested that poor communication between physicians and cancer patients might lead to patients' dissatisfaction. Thus, these patients are more likely to seek alternative methods for their treatment outside the conventional treatment. In other words, it is argued that if patients could better communicate with their care physicians, then it would be possible to receive enough information on the progress of their disease and treatment, and therefore there would not be a ground for seeking alternative methods, or if they still felt it were necessary, they would consult with their physicians about the risks and benefits of complementary therapies.

## 5. Conclusion

CAM use is common among cancer patients on treatment with radiation therapy in Thailand. The patients with a higher income were more likely to use CAM than those with a lower income. However, the expense of CAM use is relatively expensive when compared to their income. Most of the patients expect to be improved from suffering symptoms of cancer and medical treatment, but only half of the patients experienced the benefit of CAM. The majority of patients did not disclose their use of CAM to their doctors because they felt that it was not necessary for doctors to know. This finding might suggest that there were some communication gaps between the clinicians and their patients. We recommend that all clinical oncologists should be concerned and ask every patient about the use of CAM as a routine practice.

## Figures and Tables

**Table 1:** Patient characteristic of CAM users and non-CAM users.

Characteristics	CAM users (%) 151 (60.9)	Non-CAM user (%) 113 (39.1)	*P* value
*Sex *			0.254
Male	47 (56)	37 (44)	
Female	104 (63.4)	60 (36.4)	
*Mean age*	53.7 yrs	54.3 yrs	0.728
*Marital status *			0.155
Single	21 (63.6)	12 (36.4)	
Married	100 (57.1)	75 (42.9)	
Widowed/divorced	28 (73.7)	10 (26.3)	
*Education status *			0.327
Primary school or lower	61 (55.4)	49 (44.6)	
Secondary/vocational school	43 (65.2)	23 (34.8)	
Bachelor or higher	46 (66.7)	23 (33.3)	
*Occupation *			0.374
Unemployed/retired/housewife	61 (55.4)	49 (44.6)	
Employee	28 (71.8)	11 (28.2)	
Government official	25 (71.4)	10 (28.6)	
Business owner	18 (58.1)	13 (41.9)	
Agriculturist	17 (56.7)	13 (43.3)	
*Income (USD/month) *			0.001*
Less than 166	38 (46.3)	44 (53.7)	
167–333	32 (60.4)	21 (39.6)	
334–666	40 (71.4)	16 (28.6)	
More than 666	41 (71.9)	16 (28.1)	
*Cancer type *			0.019*
Breast	38 (61.29)	24 (38.71)	
Genitourinary	36 (67.9)	17 (32.1)	
Head and neck	31 (60.8)	20 (39.2)	
Gastrointestinal	8 (34.8)	15 (65.2)	
Lung	11 (78.6)	3 (21.4)	
Brain	13 (86.7)	2 (13.3)	
Others	7 (41.2)	10 (58.8)	
Not know/uncertain	7 (53.8)	6 (46.2)	
*Cancer stage *			0.761
Stage I	33 (58.9)	23 (41.1)	
Stage II	40 (58.8)	28 (41.2)	
Stage III	29 (51.8)	27 (48.2)	
Stage IV	12 (50)	12 (50)	
Do not know/uncertain	32 (86.5)	5 (13.5)	

**Table 2:** Types of complementary and alternative medicine used by patients (*n* = 151).

Type	Frequency (%)
Diet & nutrition	
Food/vitamin supplement	86 (56.9)
Dietary adjustment	75 (49.7)
Vegetarian food	25 (16.6)
High dose vitamin C	14 (9.3)
Physical body/relaxation	
Massage	34 (22.5)
Aromatherapy	23 (15.2)
Detoxification	20 (13.3)
Electromagnetic therapy	4 (2.6)
Acupuncture	3 (2.0)
Mind-body	
Meditation	64 (42.4)
Yoga	8 (5.3)
Tai chi	6 (4.0)
Yorae	5 (3.3)
Herbal medicine	47 (31.1)
Spiritual therapies	17 (11.3)

## Appendix

**Table:**

I.D.........
Please indicate your answers in the spaces provide below. (If you do not want to answer a question, please leave it blank)
(1) Diagnosis
□ Liver cancer
□ Lung cancer
□ Skin cancer
□ Lymphoma
□ Brain cancer
□ Breast cancer
□ Gastric cancer
□ Esophageal cancer
□ Colorectal cancer
□ Uterine cancer
□ Cervical cancer
□ Prostate cancer
□ Head and neck cancer
□ Nasopharyngeal cancer
□ Laryngeal cancer
□ Bladder cancer
□ Bone cancer
□ I do not know
□ Other (please specify)…………..
(2) Stage of cancer
□ (1)
□ (2)
□ (3)
□ (4)
□ (5) I don't know
(3) Age, yr...................
(4) Sex
□ (1) Male
□ (2) Female
(5) Highest level of education completed
□ (1) None
□ (2) Primary school
□ (3) High school
□ (4) College
□ (5) Professional degree
□ (6) Other (please specify).............................
(6) What is your religion?
□ (1) Bhudism
□ (2) Muslim
□ (3) Christian
□ (4) Other (please specify)………………
(7) Marital status
□ (1) Single
□ (2) Married
□ (3) Widowed/divorced
(8) Employment status
□ (1) Employed (full time)
□ (2) Employed (part time)
□ (3) Employed but on medical leave/disability
□ (4) Self-employed
□ (5) Other (please specify).................
(9) What is your monthly income?
□ (1) No income
□ (2) Less than 1,500 USD
□ (3) 1500–3500 USD
□ (4) 3500–7000 USD
□ (5) 7000–10000 USD
□ (6) 10000 USD
(10) What treatment have you had for your cancer?
□ (1) Chemotherapy
□ (2) Radiation therapy
□ (3) Surgery
□ (4) Biological or targeted therapy
(11) Do you currently use any supplements or alternative therapies or have you used these in the past
□ (1) Yes (then proceed to question 12)
□ (2) No
(12) Please check all that you currently use or have you used these in the past (please check all that apply)
□ (1) Food/vitamin supplement
□ (2) Dietary adjustment
□ (3) High dose vitamin C
□ (4) Vegetarian diet
□ (5) Detoxification
□ (6) Acupuncture
□ (7) Massage
□ (8) Aromatherapy
□ (9) Electromagnetic therapy
□ (10) Spiritual therapies
□ (11) Herbal medicine
□ (12) Meditation
□ (13) Tai chi
□ (14) Yoga
□ (15) Yorae
□ (16) Other (please specify)..............
(13) How did you learn about these supplements or alternative therapy? (check all apply)
□ (1) Family members
□ (2) Friends
□ (3) Personal knowledge
□ (4) Doctor
□ (5) Books/Magazines/TV/Radio
□ (6) Other cancer patients
□ (7) Other (please specify).................
(14) When using these supplements or alternative therapies, have they benefited you?
□ (1) No effect
□ (2) Good effect
□ (3) Moderate effect
□ (4) Uncertain
(15) When using these supplements or alternative therapies, have you experienced unpleasant side effects?
□ (1) Yes, specify..........................
□ (2) No
□ (3) Uncertain
(16) About how much money have you spent on supplements or alternative therapies?..........USD/month
(17) Have you told your doctor about these supplements or alternative therapies?
□ (1) Yes because
□ (1.1) Doctor asked
□ (1.2) Doctor should know
□ (1.3) Wanted to know the doctor's opinion
□ (1.4) Other (please specify)...............................
□ (2) No because
□ (2.1) Doctor did not ask
□ (2.2) It was not necessary for doctor to know
□ (2.3) Doctor would disapprove
□ (2.4) Other (please specify)....................................
(18) If you told your doctor, what was his/her reaction? (check all apply)
□ (1) Doctor in favor
□ (2) Doctor opposed
□ (3) Doctor do not offer opinion
□ (4) Other (please specify)....................
.....................
Thank you
